# Dynamic Transitions of Pediatric Sepsis: A Markov Chain Analysis

**DOI:** 10.3389/fped.2021.743544

**Published:** 2021-10-01

**Authors:** Sherry L. Kausch, Jennifer M. Lobo, Michael C. Spaeder, Brynne Sullivan, Jessica Keim-Malpass

**Affiliations:** ^1^School of Nursing, University of Virginia, Charlottesville, VA, United States; ^2^Center for Advanced Medical Analytics, University of Virginia, Charlottesville, VA, United States; ^3^Department of Public Health Sciences, University of Virginia, Charlottesville, VA, United States; ^4^Department of Pediatrics, Division of Pediatric Critical Care, University of Virginia School of Medicine, Charlottesville, VA, United States; ^5^Department of Pediatrics, Division of Neonatology, University of Virginia School of Medicine, Charlottesville, VA, United States

**Keywords:** Markov chain, illness transition states, transition matrix, Shannon entropy, entropy, stochastic modeling, trajectory analysis

## Abstract

Pediatric sepsis is a heterogeneous disease with varying physiological dynamics associated with recovery, disability, and mortality. Using risk scores generated from a sepsis prediction model to define illness states, we used Markov chain modeling to describe disease dynamics over time by describing how children transition among illness states. We analyzed 18,666 illness state transitions over 157 pediatric intensive care unit admissions in the 3 days following blood cultures for suspected sepsis. We used Shannon entropy to quantify the differences in transition matrices stratified by clinical characteristics. The population-based transition matrix based on the sepsis illness severity scores in the days following a sepsis diagnosis can describe a sepsis illness trajectory. Using the entropy based on Markov chain transition matrices, we found a different structure of dynamic transitions based on ventilator use but not age group. Stochastic modeling of transitions in sepsis illness severity scores can be useful in describing the variation in transitions made by patient and clinical characteristics.

## 1. Introduction

The hallmark of sepsis, organ dysfunction resulting from a dysregulated host response to infection, often requires ICU-level interventions for physiologic organ support (1). In the United States, more than one-third of children who die in tertiary care Pediatric Intensive Care Units (PICUs) have severe sepsis (2). In addition, survivors of sepsis have increased lengths of hospitalizations and are at risk of long-term complications (3, 4). Despite growing research in this area, sepsis remains a significant cause of pediatric morbidity and mortality. Better targeting of sepsis interventions following diagnosis may result in improved outcomes, yet we remain limited in our ability to target sepsis interventions to individual patients.

Recently, machine learning techniques have been employed to develop models that predict future clinical deterioration, including sepsis. Continuous electrocardiogram data from bedside monitors, vital signs, laboratory values, and clinical assessment findings in the electronic health record can be analyzed to identify patients at rising risk of sepsis, prior to overt clinical signs. Predictive models exist that were developed as time series measures of changing risk based on clinical variables that detect physiological changes with illness (5–9). The utility of such continuous predictive analytics is intuitive: novel monitoring to alert busy clinicians to a change in the patient so diagnosis and treatment can occur early. Sepsis prediction models have led to improved outcomes in the neonatal ICU and were associated with lower rates of septic shock in an adult ICU (5, 6). While risk scores from predictive analytic models have been used to provide early warning to clinicians, less research has focused on the use of this innovative derivation of complex physiologic data to characterize illness states (10).

A risk score incorporating an assessment of the illness trajectory over a period of time can capture the complex courses of patients with sepsis. Because risk scores from predictive analytics can be assessed serially, the integrated risk score also has potential utility as an index of acuity, as a proxy of responsiveness to therapy (i.e., a response biomarker), and as a way to monitor the overall trajectory of illness (11–13). By assessing the integrated continuous predictive analytic across the patient trajectory in the form of a highly-dimensional time series, we have the opportunity to pursue patient-centered modeling. The goal of patient-centered modeling is to classify individuals into distinct groups or categories based on individual response patterns over time (14). By approaching pediatric sepsis in this way, we also have the opportunity to determine if there are sub-types of trajectories, in other words—children who may have a more favorable or unfavorable early response to therapy and associated prognosis. Additionally, the majority of sepsis research has focused on early diagnosis, initiation of goal-directed therapy, and characterizing phenotypes of sepsis at the time of diagnosis (15, 16). The immediate post-diagnosis trajectory has received less attention. However, the time period following sepsis diagnosis is clinically relevant due to the persistent need to assess appropriate responsiveness to therapy and escalate or de-escalate care to improve outcomes (11, 13, 17). Thus, the temporal characteristics of illness state transitions and patterns of recovery may be essential features in understanding the illness course and for assessing how interventions affect sepsis recovery.

Markov chain models can provide insights into disease dynamics (18, 19). Markov models provide interpretable, clinically relevant metrics, such as probabilities of transitioning between illness states and the expected time required to move from one illness state to another illness state (20, 21). Temporal characteristics and state-based transitions have been used in statistical models to characterize the burden of disease and the impact of specific therapeutic interventions on recovery (22). We use Markov chain modeling to evaluate the dynamic transitions in illness states following sepsis in PICU patients in order to quantify the early illness trajectory. We use risk scores generated from a sepsis prediction model to define illness states. Our first aim was to characterize a Markov chain transition matrix for a cohort of PICU patients meeting sepsis criteria in the early 72 h time period following diagnosis. Our second aim was to further describe Markov chain transition matrices stratified by clinical characteristics (e.g., mechanical ventilation). We used a measure of entropy to characterize the differences between stratified matrices quantitatively. Finally, we examined the sequence of transitions among illness states to determine how much time was required to reach a target illness state, given an initial illness state, in a probabilistic fashion.

## 2. Methods

The University of Virginia Institutional Review Board approved this retrospective cohort study.

### 2.1. Study Design

Spaeder and colleagues developed a sepsis prediction model for use in the PICU population at the University of Virginia Children's Hospital (7). The model produced, for each patient, a continuous score that is the fold increase in the risk of developing sepsis in the following 24 h. We used the output from this model to represent the trajectory following sepsis diagnosis instead of only representing the trajectory leading to sepsis. This model development study included all admissions to the 17-bed PICU from December 2013 to May 2016. The study authors recorded demographic information, including age, length of hospitalization, length of time on a ventilator, and mortality (assessed as all in-hospital mortality), during the trial. Archived data were available for 1,711 unique admissions involving 1,425 patients. The model was trained only on sepsis events that occurred in the PICU setting. Sepsis present on admission or occurring on the acute care ward were not included in the model development.

We used the risk scores produced by the prediction model to construct matrices of the probabilities of transitioning from any given illness state to another within a 30-min period in the 3 days following cultures for suspected sepsis. We further characterized these transition matrices using Shannon entropy (23). We examined: (1) transition matrices for the cohort of admissions where sepsis occurred, (2) simulations of illness trajectories, (3) transition matrices stratified by different clinical characteristics, and (4) mean first passage times across the stratifications. Mean first passage times present the number of time steps required to reach a target illness state from an initial illness state. Analyses were performed using R studio version 3.6.2. The R package markovchain was used to calculate transition matrices and mean passage times. The simulation of trajectories was implemented in Python.

### 2.2. Description of the Sepsis Prediction Model

#### 2.2.1. Data Inputs to the Predictive Model

Inputs to the model algorithm include (1) continuous cardiorespiratory monitoring waveforms (three leads of ECG sampled at 240 Hz and pulse plethysmography and invasive blood pressure tracings at 120 Hz), (2) continuous cardiorespiratory vital signs (heart rate, respiratory rate, peripheral oxygen saturation, invasive blood pressure, ventilator measured respiratory rate, and sample-and-hold non-invasive blood pressure) sampled at 0.5 Hz, (3) clinician-entered vital/clinical signs (oxygen saturation, temperature, Glasgow coma scale, and fraction of inspired oxygen), (4) laboratory measurements (serum sodium, potassium, chloride, bicarbonate, blood urea nitrogen, creatinine, glucose, calcium, white blood cell count, hematocrit, platelet count) and BUN-to-creatinine ratio, and (5) clinical covariates (age, male gender, presence of an arterial line, and the presence of mechanical ventilation) (7). Cardiorespiratory dynamics measured from the continuous cardiorespiratory monitor, unseen by clinicians, were calculated as described by Moss et al. (24). These 16 measures were calculated in 30 min windows every 15 min. Intermediate features, censored when the values were more than 24 h old for vital signs and 48 h old for laboratory values, were combined with continuously obtained features using sample-and-hold.

#### 2.2.2. Model Development

The model was developed for two use cases: (1) to provide continuous risk estimates and (2) to provide sepsis screening alerts. A random forest model was developed on 100% of the hospital admissions and validated using cross-validation. Missing data was imputed with median values. Leave-one-out cross-validation was used to predict risk. The model output represents the fold increase in risk that a child will be diagnosed with sepsis in the following 24 h compared with the average risk of sepsis. The area under the receiver operating characteristic curve (AUC) was calculated to evaluate model performance. Confidence intervals were calculated based on 200 bootstrap runs resampled by admission. The model had an AUC of 0.750 (95% CI: 0.708–0.809). For comparison, the AUC for SIRS, with a 12-h prediction window, was 0.663 (95% CI: 0.632–0.695).

#### 2.2.3. Sepsis Definition

Sepsis events were established based on the 2005 International Pediatric Sepsis Consensus Conference criteria (25). Episodes of sepsis were defined as (1) the presence of systemic inflammatory response syndrome (SIRS) and (2) suspected or proven invasive infection caused by any pathogen. For every patient who had a blood culture order, each chart was individually reviewed by a clinician to establish the time of the sepsis event (i.e., the time of blood culture order or time of blood culture collection, whichever came first) in cases where a patient met SIRS criteria in the 12-h window preceding the culture and received antibiotics in the 6-h window following cultures.

#### 2.2.4. Description of the Data

Sepsis occurred in 157 of the 1,711 PICU admissions. In admissions with multiple sepsis events, only the first event was included in this analysis. The model generated risk scores every 15 min for each patient. To account for the fact that the model used the preceding 30 min of continuous cardiorespiratory data to generate risk scores, this study used scores every 30 min. Additionally, evaluating illness state changes every 30 min has a desirable clinical correlate to the frequent clinician monitoring that occurs in the PICU setting. Nonconsecutive risk scores occurred in 71 observations and were removed from the analysis (0.3% of the total data). The remaining 18,666 scores were adjacent 30-min score pairs. All risk scores were labeled with the corresponding time in minutes following the sepsis diagnosis. Actual times were not included; times following sepsis diagnosis for each patient were used to obtain scores in the appropriate period following sepsis and in the correct time order for this Markov chain implementation.

### 2.3. Markov Chain Assumptions

A system must have a set of distinct states and identifiable transitions among those states to be modeled as a discrete-time Markov chain (26). The transition probabilities among the identified states can be estimated for each possible transition based on the observed data at specified time intervals. A first-order Markov chain assumes behavior in the future can be predicted using only the current state. Therefore, Markov chains are considered to be “memoryless.” This has a desirable clinical correlate. While clinicians often have knowledge of prior medical history, cumulative treatment burden, physiological trends, and past responsiveness to therapy, there are many times during a critical illness when clinicians are making treatment decisions based on the current physiological state. However, Markov chains can be constructed to maintain a memory effect by accounting for prior state transitions. For example, in a second order Markov chain, each observation is influenced by the two previous observations. We constrain our examination to only the first-order Markov chain.

We assume transition probabilities are independent of time. We examine the 72-h period following cultures obtained for sepsis as the time of interest in the course of sepsis illness. However, illness transition probabilities may be conditional on time. Clinically, we can see that illness resolution is not guaranteed in the days following sepsis. We examine if the assumption of time-independent probabilities holds by comparing the transition probabilities of one week to those of 3-day periods. Finally, this is a population-level analysis rather than an individual-level analysis. Transition probabilities are aggregated across all patients. By stratifying groups based on specific characteristics, we will partially address this limitation.

### 2.4. Markov Chain Construction

Risk scores generated from the model are the fold-increase in sepsis relative to the average risk of sepsis in the study population. A relative risk of 1.0 indicates the average risk while 2.0-fold indicates twice the average risk. Risk scores ranged from 0 to 8. To create clinically meaningful, discrete illness states, the scores were binned into four groups (0–3). The lowest illness state, 0, has risk scores in the range [0,1]. Illness state 1 has risk scores in the range [1,2], representing those with an increased risk. Risk scores in the range [2,3] compose illness state 2, and represent a higher illness state than the preceding states of 0 and 1. The highest risk illness state, 3, contains all scores 3 or higher.

With four illness states, there are 16 possible transitions and associated transition probabilities. The transition matrix is created by row, that is the probabilities in a row sum to one. Specifically, the number of transitions from the initial illness state to the next illness state are counted and inserted into the corresponding cell in the transition matrix. Then, each cell in the row is divided by the sum of transition counts for that row. Transition matrices were calculated in two ways: (1) as non-absorbing matrices where only the illness states are considered as possible transient states, and (2) including death as an absorbing state in addition to the four transient illness states. This matrix has five rows, with the fifth row representing the absorbing state of death.

### 2.5. Developing the “Entropy Matrix”

Entropy can be considered as a measure of disorder within a system (27). The Shannon entropy of a random variable is (23):


(1) $$\begin{array}{l} H=-\sum p\left(x\right)log\;p\left(x\right) \end{array}$$

Distributions that are peaked around only a few values will have low entropy relative to more uniform distributions. We will calculate the Shannon entropy of the distribution of transition probabilities by recalculating the transition matrix. In the transition matrix, all of the rows are probability distributions and sum to one. We create an “entropy matrix” where matrix cells sum to one. In this matrix the number of transitions observed in each of the matrix cells is divided by all observed transitions. We use natural logarithms to define entropy.

### 2.6. Simulating Trajectories

An illness trajectory can be simulated from a Markov chain based on a starting state and probabilities from the transition matrix. After obtaining the transition matrix of probabilities and selecting an initial illness state, a multinomial distribution can be generated based on the corresponding row of probabilities in the transition matrix.

### 2.7. First Passage Times

In this context, the first passage times show how much time it would take to reach a destination illness state for the first time from a given initial illness state using the probabilities in the transition matrix. For each possible initial illness state, the number of time steps required to reach the target destination state is calculated.

## 3. Results

### 3.1. Characteristics of Patients

Demographic information of the cohort is given in Table 1. Sepsis occurred in 157 PICU admissions involving 140 individual patients. In the 3 days following sepsis there were 18,666 observed illness states. Twenty-seven of the admissions ended with the death of a patient, with 16 of those deaths occurring within the 3 days following sepsis. One hundred and twenty-nine admission with sepsis required mechanical ventilation, with a median duration of ventilation of 207 (IQR: 78–638) hours. The median age of the cohort was 1.2 years.

**Table 1:** Sociodemographics of the study cohort.

**Variable**	**Levels**	**Value**
Illness state (No. of observations)		18,666
	0	3,878
	1	6,433
	2	4,129
	3	4,226
Age (mean, SD)		4.2, 5.3
Age groups (No. of sepsis events)	0–1	78
	Over 1	79
Sex (No. of sepsis events)	Male	80
	Female	77
Survived (No. of sepsis events)	Survived	130
	Deceased	27
Ventilator Groups (No. of sepsis events)	Ventilated	129
	No ventilation	28

### 3.2. Characterizing the Transition Matrix

**Figure 1:**
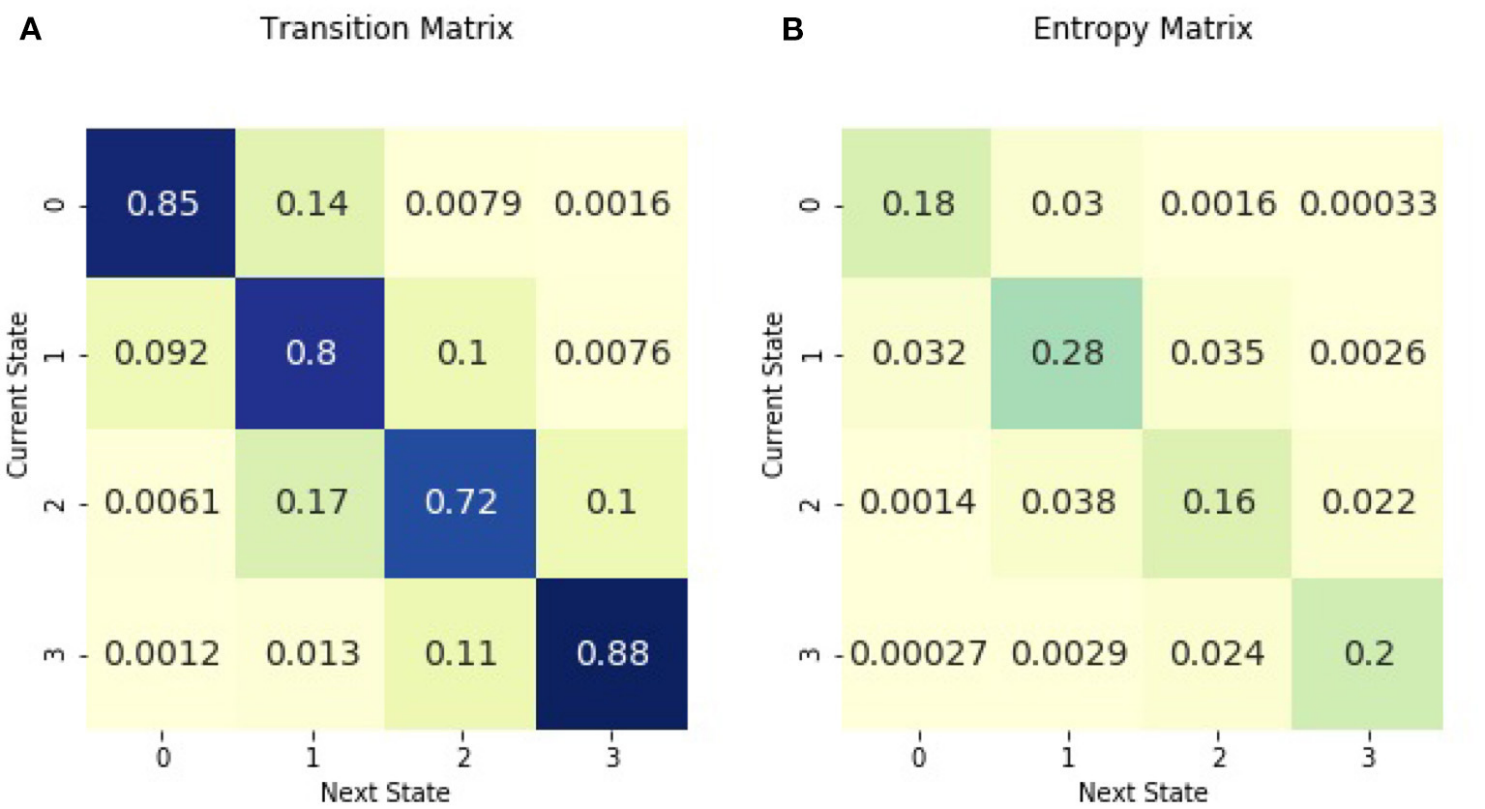
Across all patients in the 3 days following sepsis, **(A)** is the transition matrix. The matrix shows the probability of transitioning from a current illness state, denoted by rows, to the subsequent illness state, denoted by columns. **(B)** is the entropy matrix. The probabilities in the entropy matrix are normalized from all initial illness states. Thus, values in the entropy matrix indicate the density of the observed transitions. The visual difference between the transition and entropy matrices arises from the fact that the the row values sum to one in the transition matrix while all the cell values sum to one in the entropy matrix. The figure may be interpreted as follows. The darkest cell in the entropy matrix is in the second row and the second column of **(B)**, where 28% of observed transitions occurred. The corresponding cell in **(A)** signifies that there is an 80% chance of remaining in illness state 1 for patients currently in illness state 1.

Figure 1 shows the transition matrix for the entire cohort of children in the 3 days following sepsis. Transition probabilities ranged from 0.88 to <0.01. The highest transition probabilities were along the diagonal, with patients most likely to remain in the same illness state. The Shannon entropy for the entropy matrix of transitions was 1.96. For a point of reference, the minimum entropy value possible is 0, characterizing a matrix with a probability of one in one cell and a probability of zero in all remaining cells. The maximum entropy for a 16-cell matrix is 2.77, representing the case when the probabilities are uniformly distributed among the cells.

### 3.3. Simulating Trajectories

**Figure 2:**
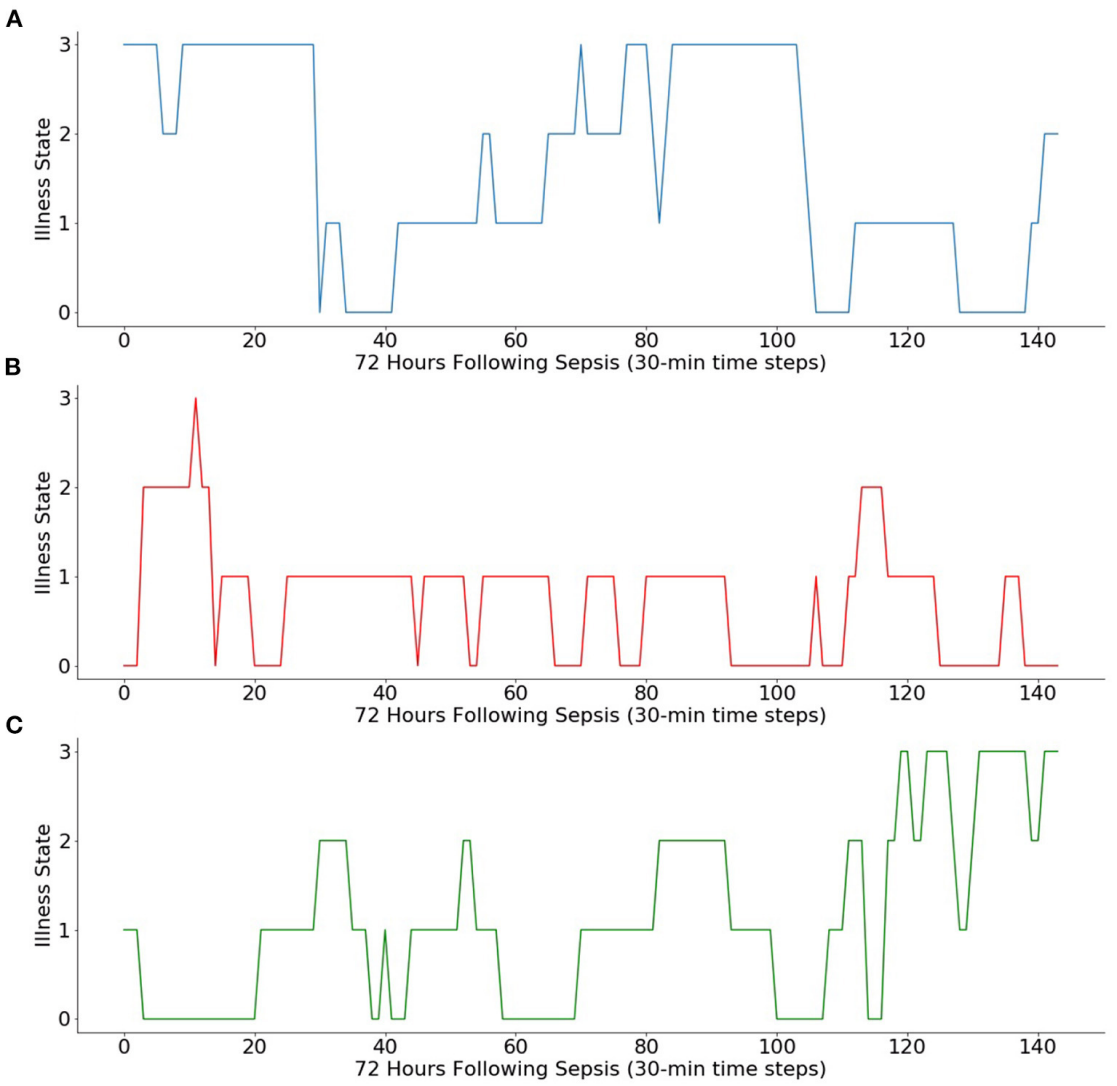
Simulations of Markov chain iterations from three initial illness states. Using the Markov chain, 3 days of transitions (i.e., 144 sequential 30-min transitions) are simulated from **(A)** initial illness state of 3, **(B)** initial illness state of 0, and **(C)** initial illness state of 1.

The transition matrix can be used to simulate Markov chain iterations from initial illness states, as shown in Figure 2. Simulated trajectories offer a probabilistic method to examine how illness states may vary between high and low levels of illness following diagnosis regardless of the initial illness state.

### 3.4. Characterization of Stratified Transition Matrices and Entropy Matrices

**Figure 3:**
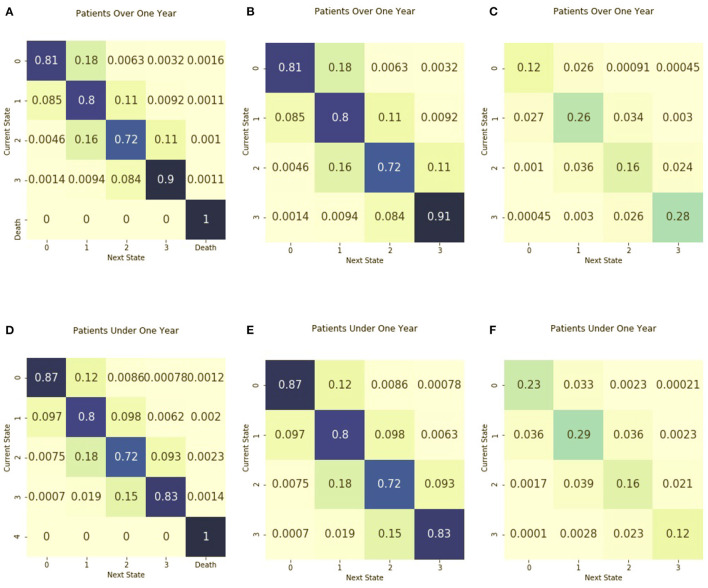
Transition matrices stratified by age group. Separate transition matrices were created for **(A)** patients older than 1 year of age and including death as an absorbing state **(B)** patients older than 1 year of age and including only transient illness states, and **(C)** the entropy matrix for patients older than 1 year of age. The transition matrices are shown for **(D)** patients from birth to age one, including death as an absorbing state **(E)** patients from birth to age one with only transient illness states, and **(F)** the entropy matrix for patients from birth to age one.

Figure 3 includes the absorbing and non-absorbing transition matrices stratified by age and the corresponding entropy matrices. When stratified by age less than or greater than 1 year, the transition matrix is similar for both age groups. The entropy of the two matrices is also similar. The entropy of the matrix for patients under 1 year 1.95, similar to the entropy of 1.92 for those older than 1 year. The absorbing transition matrix shows that patients died in both age groups and the transitions to death occurred across all illness states.

**Figure 4:**
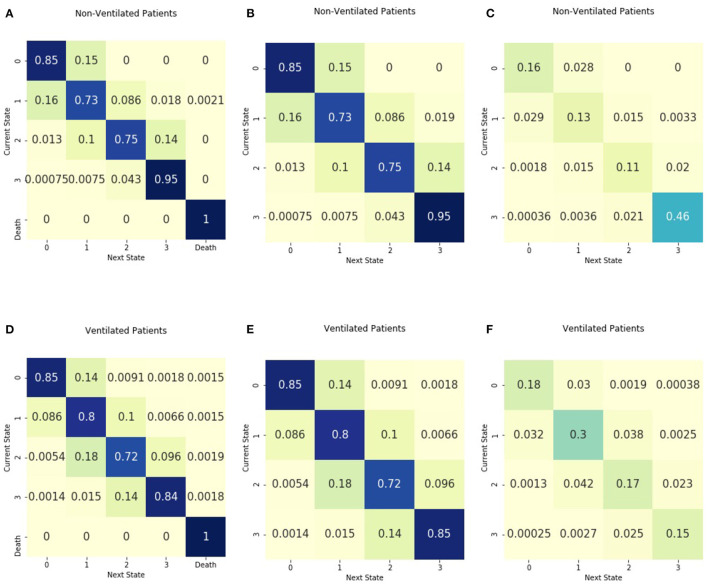
Transition matrices stratified by ventilator use. Separate transition matrices were created for **(A)** patients without mechanical ventilation and including death as an absorbing state **(B)** patients without mechanical ventilation with only transient illness states, and **(C)** the entropy matrix for patients without mechanical ventilation. The transition matrices are shown for **(D)** patients requiring mechanical ventilation, including death as an absorbing state **(E)** patients requiring mechanical ventilation with only transient illness states, and **(F)** the entropy matrix for patients requiring mechanical ventilation.

Figure 4 includes the absorbing and non-absorbing transition matrices stratified by ventilator use and the entropy matrices. Non-ventilated patients had the greatest density of transitions in illness state 3 and had a probability of 0.95 of remaining in the highest illness state. Ventilated patients had the greatest density of transitions in illness state 1. For those in illness state 3, the probability of transitioning to a lower illness state was 0.14, greater than the 0.04 probability of transitioning to an illness state of 2 for those in the non-ventilated group. The entropy of the matrix for ventilated patients is 1.96, higher than the entropy of 1.70 for non-ventilated patients. The absorbing transition matrix shows that most patients who died were ventilated and that the transition to death occurred across all illness states.

### 3.5. First Passage Time to Target Illness States

**Figure 5:**
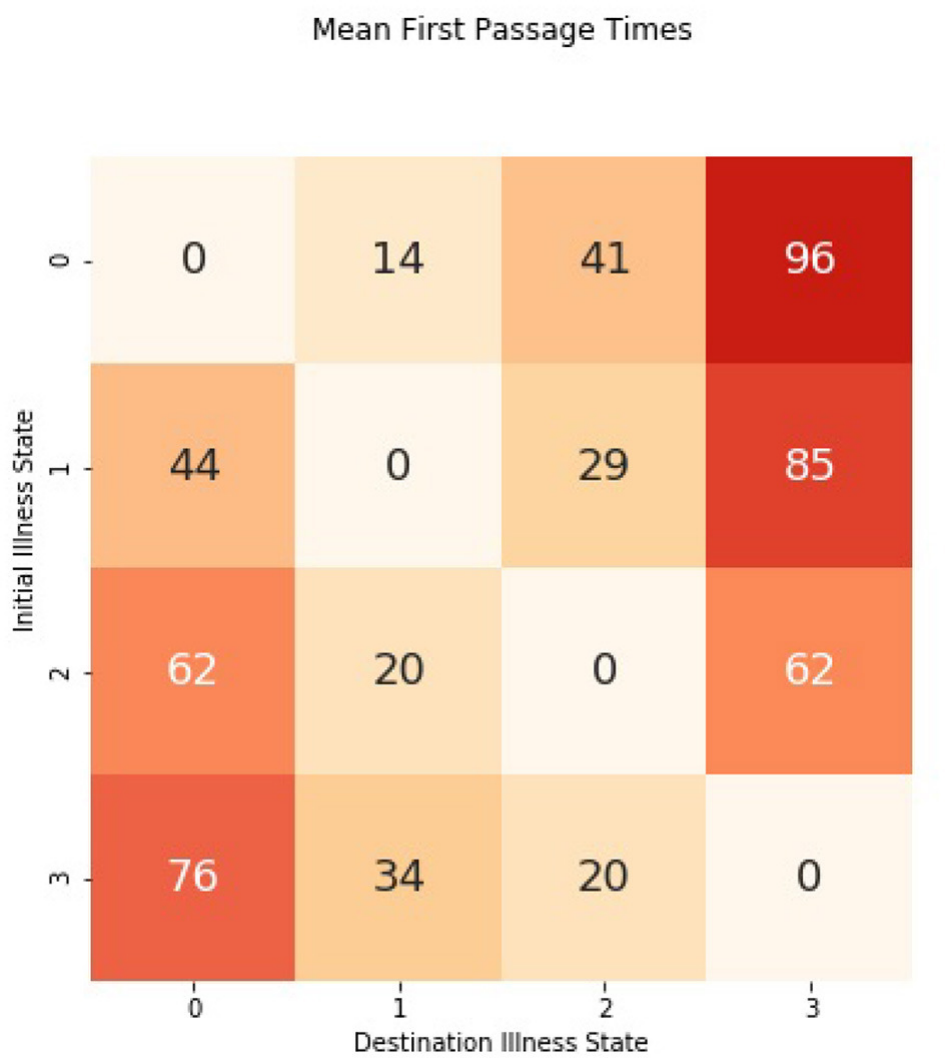
Mean first passage times from initial illness states to destination illness states. For each possible initial illness state, denoted by rows, the amount of time (in hours) required to reach the destination illness state in shown. The 0 s along the diagonal indicate that the number of steps to reach the same state were not calculated.

**Figure 6:**
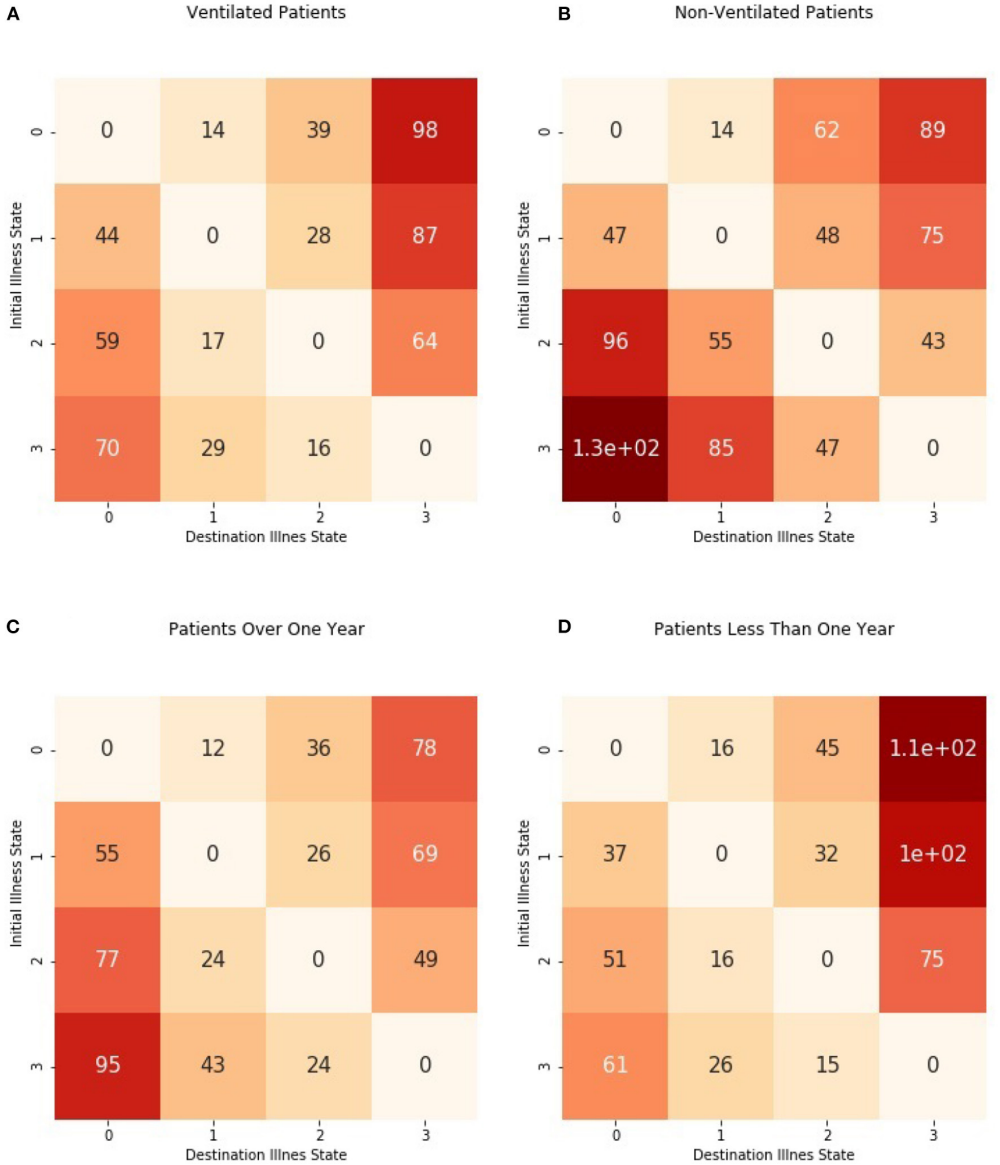
Mean first passage times from initial illness states to destination illness states stratified by ventilator use and age group. Times were estimated separately for **(A)** patients requiring mechanical ventilation and **(B)** patients without mechanical ventilation as well as **(C)** patients older than 1 year and **(D)** patients from birth to 1 year.

We examined mean first passage times, or how much time was required for a patient to move from an initial illness state to a target illness state. Figure 5 shows the first passage times (in hours) from each possible initial illness state to each possible destination illness state. We did not consider the times required to re-enter the same illness states, and the time is denoted as 0 in the matrix. Figure 6 shows passage times stratified by ventilator use and age.

### 3.6. Assumption Testing

We assumed a discrete-time Markov chain would characterize illness state transitions in the time immediately following sepsis. We tested this assumption by examining different periods following sepsis. Transition matrices were examined for a period of 7 days in addition to a period of 3 days following sepsis. The transition matrix of a 3-day period modeled the population-level illness state transitions better than a seven-day period and was used in this analysis. Supplementary Figures 1–4 display model fit for each illness state. Supplementary Figure 5 shows the transition matrices based on a 7-day period. We also examined the effect of different binning of illness states on the transition matrix. Transition matrices were examined using illness states binned into five and six categories, without changes in the structure of the matrices. The number of illness state observations we not equal among the groups in the four-bin structure we used in this analysis. The majority of illness state observations were in state 1 (see Table 1). Therefore, an additional four-bin structure was examined where there were an equal number of total observations over the four illness states (see Supplementary Figure 6) without a change in the structure of the transition matrix.

## 4. Discussion

We studied the trajectories of illness severity indices in a cohort of children admitted to the PICU. We undertook stochastic methods to explore physiological state transitions in children in the hours following a sepsis diagnosis. When using Markov chains to model trajectories, the trajectories are defined by the probability of transitioning between states. These probabilities are presented in the transition matrices. Differences in trajectories can be seen in the differences between the transition probabilities when stratifying by ventilator status or age or other clinical factors to understand the differences in temporal dynamics of illness severity between these clinical factors. Our results demonstrate that the population-based transition matrix of sepsis illness severity scores in the hours following a sepsis diagnosis can describe a sepsis illness trajectory. Additionally, the calculation of Shannon entropy can be useful in describing the variation in transitions made across patient characteristics and clinical factors.

The transition matrices stratified by age are similar, both in terms of the probability of transitions between illness states and in the distribution of observed transitions. Shannon entropy is also similar between the two matrices. Thus, one interpretation is that the illness trajectory of sepsis is similar across ages. Also, this could speak to the performance of the prediction model when it was developed. The assessment of sepsis trajectories by age is helpful due to the vast physiological and developmental differences seen in the population of children in the PICU, ranging from neonates to young adults.

The transition matrices stratified by ventilator group suggest a difference in illness trajectory dynamics between the two groups. Those who require mechanical ventilation have a greater density of observed transitions in the lower illness states as compared with those who do not require ventilation. In the non-ventilated group, almost half of the observed transitions occur in the highest illness state. Shannon entropy is also different between these groups, with higher entropy in the transitions of ventilated patients. This could speak to the role of respiratory rate on illness severity calculations (7). There is a potential that those with mechanical ventilation would have respiratory rates within normal ranges due in part to the ventilator breathing for them. These differences between groups further highlight the need to explore sepsis disease dynamics and therapeutic intensity simultaneously.

Very little work has been focused on the critical period immediately following a sepsis diagnosis where clinicians must carefully assess responsiveness to therapy or the need to change antibiotic regimens. Further, there are very few biomarkers that are indicative of sepsis severity. The biomarkers that do exist (e.g., lactate, procalcitonin) require serial blood draws for laboratory assessment and are not obtained at the same frequency an illness severity score is accessible (28–31). Approaches that assess differences in illness dynamics that are associated with successful recovery can be used in conjunction with established biomarkers and assessments of the level of therapeutic intensity, or how much support (i.e., vasopressor requirements, use of mechanical ventilation, or extracorporeal membrane oxygenation) the child requires to maintain physiological stability (32). Understanding these patterns and variations between children who survive hospitalization and children who do not may be of immense clinical utility in this early period of sepsis, where clinical regimens may be further tailored to risk.

This analysis provides an essential first step toward future analyses utilizing Markov decision processes to optimize clinical interventions to improve illness trajectories. It also suggests the potential to use reinforcement learning in this post-sepsis diagnosis period. Other approaches to illness trajectories have used longitudinal methods for evaluating change over time, which allows for apportioning of variance as well as phenotyping or clustering approaches (15, 33). In contrast, Markov decision processes can model the sequence of interactions between clinician interventions and illness states (34). This could allow for an understanding of how clinician action affects illness transitions.

One strength of this study is in our generation of Markov chains based on empirical data. We had few missing data points and a large amount of data. We re-categorized states to examine the effect of different illness state bins on transition matrix probabilities. We note that using risk scores as measures of illness severity requires a well-calibrated model. Our study had limitations. Our analysis was limited to illness state transitions based on risk scores generated from one predictive analytic model designed for use in a single PICU. External validation of our method on a different study population is needed. Additionally, it is important to acknowledge that the most up to date pediatric definition for sepsis, the International Pediatric Sepsis Consensus Conference criteria that we employed in this study, has not been updated for 16 years. We anticipate that a future update of the pediatric criteria would employ organ dysfunction as a component of the definition, similar to the adult Sepsis-3 criteria (1). In future work, our model would need to be updated to reflect the changing sepsis criteria. Finally, modeling a system as a Markov chain requires making several assumptions, notably the limitation in the Markov property and the assumption that we chose an appropriate period to study. We examined whether these assumptions held in our data and noted that the Markov assumption has a clinical concordance in how clinicians assess patients in the ICU environment.

In conclusion, we used a discrete-time Markov chain to characterize the illness trajectory following sepsis. Pediatric sepsis is a heterogeneous disease that can result in mortality or significant morbidity and prolonged physical disability (3, 4). Using the entropy based on Markov chain transition matrices, we found a different structure of dynamic transitions based on ventilator use but not age group. Elucidating these transitions and variations in illness severity is a needed area of inquiry to understand better how to characterize children's sepsis trajectories. Studying disease dynamics through stochastic approaches offers the foundation for reinforcement learning during critical clinical decision-making periods. Future work is needed to explore the relationships between therapeutic interventions and sepsis transitions and understand the burden of illness across the entire critical care trajectory.

## Data Availability Statement

The datasets presented in this study can be found in online repositories. The names of the repository/repositories and accession number(s) can be found at: Open Science Framework https://osf.io/hng2t/.

## Ethics Statement

The studies involving human participants were reviewed and approved by the University of Virginia Institutional Review Board. Written informed consent from the participants' legal guardian/next of kin was not required to participate in this study in accordance with the national legislation and the institutional requirements.

## Author Contributions

SK and JK-M conceptualized the project. JK-M obtained grant funding for this project. SK analyzed the data and drafted the manuscript. JL, JK-M, and BS advised on analysis and interpretation of the data. JL and JK-M revised the manuscript critically. MS obtained and curated the data. All authors reviewed the final manuscript before submission.

## Funding

JK-M received funding support from the Gordon and Betty Moore Foundation grant number GBMF9048 as a Betty Irene Nurse Innovator Fellow.

## Conflict of Interest

The authors declare that the research was conducted in the absence of any commercial or financial relationships that could be construed as a potential conflict of interest.

## Publisher's Note

All claims expressed in this article are solely those of the authors and do not necessarily represent those of their affiliated organizations, or those of the publisher, the editors and the reviewers. Any product that may be evaluated in this article, or claim that may be made by its manufacturer, is not guaranteed or endorsed by the publisher.
