# Supplementary material for: Dynamic Transitions of Pediatric Sepsis: A Markov Chain Analysis
Source: Front Pediatr. 2021 Oct 1;9:743544. doi: 10.3389/fped.2021.743544 (PMC8517521; doi:10.3389/fped.2021.743544)
Supplement: Supplementary file 1 [file Data_Sheet_1.pdf]

Supplementary Material

1 SUPPLEMENTARY TABLES AND FIGURES

1.1 Supplementary Terms and Definitions

Markov chain - A Markov chain is one type of stochastic model where the probability of each event depends only on the previous event. For a system to be modeled as a Markov chain, the system must have a set of distinct states and identifiable transitions among those states. The system must be observed repeatedly over set time intervals. Then, based on the observed data at each time interval, the transition probabilities among the identified states can be estimated. To model an illness trajectory using a Markov chain, we are not required to know the underlying mechanisms of the system. We model the trajectory as a series of transitions between illness states.

Illness states- Markov chains require the definition of discrete states. We defined discrete illness states ranging from 0 to 3 based in the scores produced from a prediction model. Higher illness scores were considered to represent greater illness.

Stratification by age and ventilator use- We present a population-level view of the sepsis trajectory based on Markov chain modeling. To examine differences in sepsis trajectories, we present two sub-populations of the cohort based on clinical characteristics. First, we examine the sepsis trajectory of patients who require mechanical ventilation as compared to those patients who do not require ventilation. We also compare patients less than one year of age with those over one year of age. We present these two stratifications with the goal of observing differences between the trajectories (defined by the probabilities in each transition matrix).

Transition matrix- Based on the observed state transitions, a corresponding matrix of transition probabilities can be calculated. Each cell in the transition matrix represent a probability. These probabilities describe the evolution of the system, probabilistically, from state to state over time. The rows of the transition matrix correspond to the state in time t (i.e., the initial illness state) and the columns correspond to the state at time t + 1 (i.e., at the next time step).

Mean first passage times- Mean first passage times present the number of time steps required to reach a target illness state from an initial illness state.

Shannon entropy- Claude Shannon introduced the concept of information entropy in his 1948 paper, A mathematical theory of communication. In information theory, the entropy of a random variable can be considered as the average level of surprise or uncertainty. The measure of information content depends on the probability distribution.

Sepsis prediction model- We based our illness states on the risk scores generated from a sepsis prediction model. The model was designed to function as both a continuous measure of illness and as a predictor of sepsis. Details about the model beyond what is described in the methods can be found in reference [21].

1.2 Figures

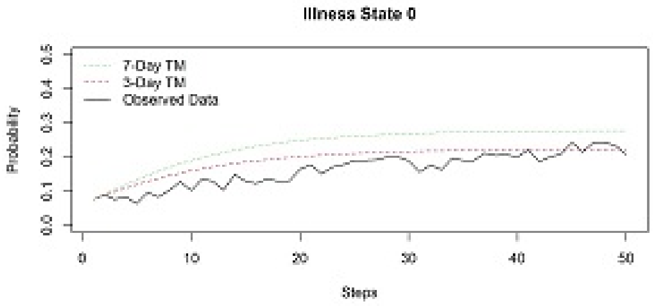

Figure S1. The black line shows the proportion of the cohort in state 0 at each of the first 50 time steps (i.e., over the first 25 hours following sepsis). The red line is the expected proportion of the cohort in state 0 based on the starting distribution and the transition probabilities in the matrix based on observations from the first 3 days following sepsis. The green line is the proportion of the cohort in state 0 based on the probabilities in the 7-day transition matrix.

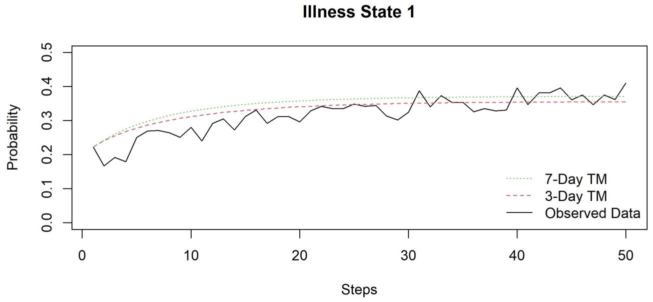

Figure S2. The black line shows the proportion of the cohort in state 1 at each of the first 50 time steps (i.e., over the first 25 hours following sepsis). The red line is the expected proportion of the cohort in state 1 based on the starting distribution and the transition probabilities in the matrix based on observations from the first 3 days following sepsis. The green line is the proportion of the cohort in state 1 based on the probabilities in the 7-day transition matrix

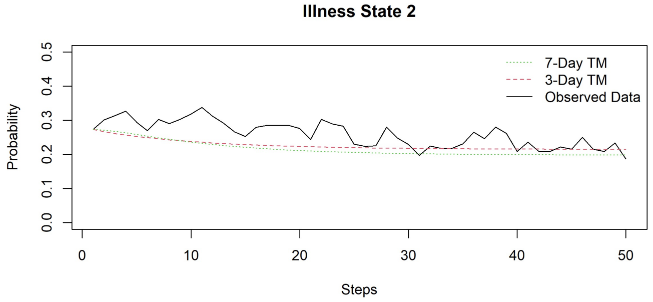

Figure S3. The black line shows the proportion of the cohort in state 2 at each of the first 50 time steps (i.e., over the first 25 hours following sepsis). The red line is the expected proportion of the cohort in state 2 based on the starting distribution and the transition probabilities in the matrix based on observations from the first 3 days following sepsis. The green line is the proportion of the cohort in state 2 based on the probabilities in the 7-day transition matrix

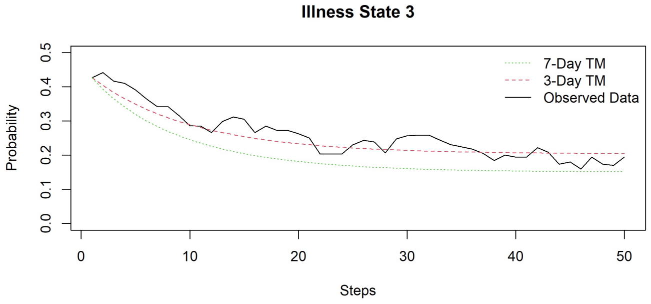

Figure S4. The black line shows the proportion of the cohort in state 3 at each of the first 50 time steps (i.e., over the first 25 hours following sepsis). The red line is the expected proportion of the cohort in state 3 based on the starting distribution and the transition probabilities in the matrix based on observations from the first 3 days following sepsis. The green line is the proportion of the cohort in state 3 based on the probabilities in the 7-day transition matrix

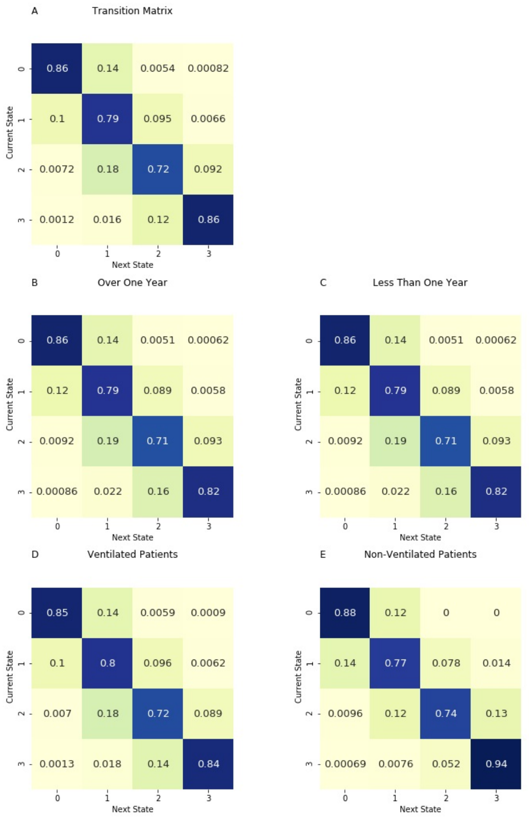

Figure S5. Transition matrices based on illness states observations for a period of 7 days following sepsis. **(A)** is the overall transition matrix, **(B)** and **(C)** are the transition matrices stratified by age, and **(D)** and **(E)** are the transition matrices stratified by ventilator use.

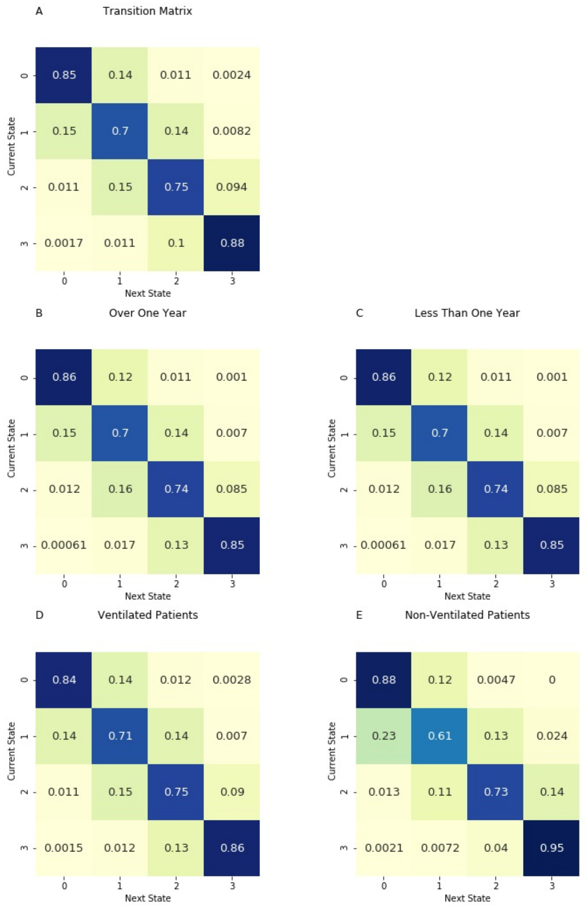

Figure S6. To investigate the effect of different illness state bins on the transition matrices, risk scores where binned into 4 illness state bins of equal probability. The distribution of all raw risk scores for all admissions over the 72 hour period following sepsis were examined and 4 bins of equal size were created. The scores for illness state 0 were in the range [0,1.11). Illness state 1 had scores in the range [1.11, 1.83). Scores in the range [1.83, 2.87) compose illness state 2, and illness state, 3, contains all scores 2.87 or higher. **(A)** is the overall transition matrix, **(B)** and **(C)** are the transition matrices stratified by age, and **(D)** and **(E)** are the transition matrices stratified by ventilator use.
